# Detecting and analyzing research communities in longitudinal scientific networks

**DOI:** 10.1371/journal.pone.0182516

**Published:** 2017-08-10

**Authors:** Valerio Leone Sciabolazza, Raffaele Vacca, Therese Kennelly Okraku, Christopher McCarty

**Affiliations:** 1 Bureau of Economic Business and Research, University of Florida, Gainesville, Florida, United States of America; 2 Department of Sociology and Criminology & Law, University of Florida, Gainesville, Florida, United States of America; Tianjin University, CHINA

## Abstract

A growing body of evidence shows that collaborative teams and communities tend to produce the highest-impact scientific work. This paper proposes a new method to (1) Identify collaborative communities in longitudinal scientific networks, and (2) Evaluate the impact of specific research institutes, services or policies on the interdisciplinary collaboration between these communities. First, we apply community-detection algorithms to cross-sectional scientific collaboration networks and analyze different types of co-membership in the resulting subgroups over time. This analysis summarizes large amounts of longitudinal network data to extract sets of research communities whose members have consistently collaborated or shared collaborators over time. Second, we construct networks of cross-community interactions and estimate Exponential Random Graph Models to predict the formation of interdisciplinary collaborations between different communities. The method is applied to longitudinal data on publication and grant collaborations at the University of Florida. Results show that similar institutional affiliation, spatial proximity, transitivity effects, and use of the same research services predict higher degree of interdisciplinary collaboration between research communities. Our application also illustrates how the identification of research communities in longitudinal data and the analysis of cross-community network formation can be used to measure the growth of interdisciplinary team science at a research university, and to evaluate its association with research policies, services or institutes.

## 1. Introduction

The recent increase in collaborative research [1,2] has led to the development of a new field, the Science of Team Science (SciTS). This field analyzes scientific collaboration, team effectiveness, and the mechanisms of team assembly using a variety of methods including network analysis [3–9]. Consistent with this framework, this paper studies the formation of communities and interdisciplinary collaborations by analyzing longitudinal collaboration networks extracted from peer-reviewed publications and awarded grants at the University of Florida (UF), a large research university comprising more than 50,000 students and 5,000 full-time faculty. We propose a method that uses community-detection algorithms to identify longitudinal research communities, i.e., sets of investigators who have been part of the same collaborative subgroup consistently over a certain number of years. We analyze the drivers of interdisciplinary collaborations that cut across different research communities using Exponential Random Graph Models (ERGM).

These models are used to quantify the effect of node-level characteristics, network topology, and the activities of a specific UF research institute on the growth of interdisciplinary collaborations in the university. The analysis focuses on a specific research institute, namely the University of Florida Clinical and Translational Science Institute (CTSI) in the UF College of Medicine, funded in part by a Clinical and Translational Science Award (CTSA) by the National Institutes of Health (NIH) National Center for Advancing Translational Sciences [10]. One of the main goals of CTSA-funded research institutes is to facilitate interdisciplinary research that aims to translate basic scientific discoveries into applications in health facilities and communities, including new treatments, clinical practices, and health policies. Existing evidence points to an important role of CTSA-funded institutes in shaping the network architecture of research universities [11–15]. While there are only 62 CTSAs, other types of cross-department and cross-college institutes are often established in universities to stimulate inter-disciplinary research. In this study, we use the UF CTSI as an example of a research institute’s effect on collaboration communities.

Scientific collaboration is often operationalized as co-authorship on peer-reviewed publications or co-participation in research grants [13–16]. This paper combines publication and grant data to obtain a more comprehensive and realistic representation of underlying collaborative relationships among scientists. Such relationships are frequently represented and analyzed using notions and methods from Social Network Analysis (SNA) and Network Science [17,18]. Networks of scientific collaboration are a crucial channel for the diffusion of information, knowledge, expertise and innovation among scientists in different disciplines [19–21]. Networks of interdisciplinary collaborations, in particular, play a central role in combining and coordinating different skill sets, models, and approaches to tackle complex problems in innovative ways and bridge disciplinary silos [1,22,23]. A growing body of evidence suggests that the highest-impact scientific work typically originates in large and cross-disciplinary teams [24–26].

SNA is a set of notions, methods and theories used to represent relations between actors and to study the role that such relations play in shaping individual and group behaviors and outcomes [27]. SNA methods have been used to analyze the structural patterns of scientific collaborations [28–30], evaluate the impact of research institutes on the scientific network of an organization [11,13–15,31] and design interventions to promote behavioral change [32, 33].

Scientific collaboration networks, both within and across institutions, are typically very large [28, 34] and comprised of many smaller collaborative subgroups. Broad institutional classifications such as institutes, departments, and colleges are unlikely to describe these groups, particularly when they include investigators from different disciplinary backgrounds and affiliations. While these units are necessary for administrative purposes, in practice they may unnecessarily constrain our understanding of collaborative behavior. By contrast, network methods for community detection [35] can help to identify collaborative subgroups as they emerge from actual interactions between scientists. Intuitively, a network community is a set of actors who densely interact with each other, but show sparser connections with the rest of the network.

The structural characteristics of scientific collaboration networks result from a variety of organizational, disciplinary, geographic, and cultural factors. Spatial proximity, homophily, transitivity, past collaboration experiences, shared funding sources, disciplinary background, and department and college affiliation all play a role in shaping the structure of scientific networks. Spatial proximity is a predictor for research collaboration, as investigators whose work locations are closer in space are more likely to engage in informal conversations, leading to professional collaborations [36–38]. In addition, analyses of homophily or assortative mixing have found that researchers are more likely to work with colleagues who are similar in discipline, tenure status, age, gender, and other background characteristics [39–40]. Transitivity, the tendency for two collaborators of the same scientist to also collaborate with each other, has been identified as another driver of scientific collaboration, due to scientists often introducing their colleagues to each other [17].

The structure of scientific networks has been analyzed in a number of papers [17,28–30,41] to detect the main topological features of these networks (e.g., small-world structures, scale-free degree distributions, community structures). The factors shaping these structures can be identified using network formation models [42]. Network formation models, including ERGMs, view a social network as the realization of a probabilistic social process in which the creation of a tie between researchers may result from different factors, including node-level attributes (e.g., the collaborators’ disciplinary background) and network topology (e.g. transitivity effects). Using network formation models, [43] and [44] assess the impact of overlapping competences and skill complementarities on the propensity to scientific collaboration among economists; [45] analyzes the effect of spatial and network proximity on the probability of collaboration between investigators working on the feminization of the labor force in Asia; [40] investigate how disciplinary affiliation affects collaborative behaviors at Washington University in St. Louis.

This paper is organized as follows. Section 2 introduces the data and the construction of the individual-level collaboration networks. Section 3 presents our methods to identify research communities and obtain community-level networks. Section 4 reports results from the analysis of the identified communities and the formation of inter-community networks. Sections 5 and 6 discuss the findings and conclude the paper.

## 2. Data

We use data on peer-reviewed publications and extramurally awarded grants of University of Florida researchers in 2013, 2014 and 2015. Publication data were obtained for all UF authors from the Thomson Reuters Web of Science, while grant records were extracted from institutional data on financial transactions between funding entities and the UF Office of Research. Web of Science data were disambiguated and stored into VIVO, a semantic-web application adopted across all colleges and departments at UF [5]. For all UF authors, VIVO creates publication IDs and links author names to unique numeric UFIDs. All the data needed to reproduce results in this paper are available at the following URL: https://github.com/CTSI-Network-Science-group/Detecting-and-analyzing-research-communities-in-longitudinal-scientific-networks.

Both publications and grants are obviously an indication of collaboration between the investigators involved, although their time cycle and the resulting data structures often differ. While in some cases groups of collaborators work on both publications and grants at approximately the same time, it is often the case that researchers collaborate on publications first, to then be able to demonstrate research results and apply for grants together; or conversely, frequently scientists obtain a grant first, which then enables them to conduct research resulting in publications. Thus at a specific point in time, for certain pairs and groups of scientists, collaboration might only occur through publications and be detectable in publication data; for others, collaboration might only take place through grants, and be visible in grant data. Dataset that combine publications and grants, therefore, offer a unique opportunity to capture multiplex and comprehensive relations of collaboration, which would remain unobserved if publications and grants were analyzed separately. However, gaining access to both publication and grant data for a given set of scientists is not common, and analyses of publication and grant collaborations taken separately prevail in the literature.

Although publication and grant collaborations can and should be analyzed separately when the interest centers on specific social processes leading to different research outcomes (e.g. publishing versus obtaining research funding), in this study we are interested in operationalizing a broader notion of scientific collaboration between individuals at a university. This paper, therefore, combines publication and grant collaborations to obtain a more comprehensive, inclusive, and realistic representation of collaborative interactions among UF scientists, compared to the single “slices” of publications and grants taken separately. Appendix A in S1 File provides more details on the different data structures implied by publications and grants.

In this study, a publication collaborative tie between researchers *i* and *j* exists in a given year *t* if one or more articles, in which *i* and *j* appear as co-authors, are published in year *t*. The number of articles co-authored by *i* and *j* in that year is stored in the data as the *weight* of that year’s publication collaborative tie between the two researchers. Similarly, a grant collaborative tie between *i* and *j* exists in year *t* if one or more grants, in which *i* and *j* appear together as PIs, multiple PIs or co-PIs, are active in year *t*. The number of such active grants is the weight of the grant collaborative tie between *i* and *j* in year *t*. We define as *union* collaboration the union of a publication and a grant collaboration. Scientists *i* and *j* have a *union* collaborative tie in year *t* if one or more articles are published in *t*, in which *i* and *j* are co-authors; or if one or more grants are active in *t*, in which *i* and *j* appear as PIs, multiple PIs, or co-PIs. The weight of the union collaborative tie is the number of such articles and grants.

In the data analyzed for this paper, year *t* is one of 2013, 2014 and 2015, the three most recent years for which complete data were available when the project started. We limited the analysis to 2013–2015 for two main reasons. In the first place, we were most confident in the completeness and accuracy of publication and grant data for the most recent three years. Available data were less complete and accurate for previous years. In the second place, and perhaps more importantly, we were most interested in patterns of recent and emergent research collaborations. It should be noted that even the most recent publication and awarded grant data have an inherent temporal lag, in that published articles and awarded grants normally follow in time, by at least several months, the actual start of a collaborative research project. While we did extract research communities based on data for more years (e.g. the most recent 5 years) in previous iterations of this project, subsequent qualitative interviews with involved investigators suggested that the resulting communities tended to capture older collaborations and to incorporate more recent research to a lesser extent. By contrast, we are interested in detecting communities that reflect more current collaborative patterns, while at the same time identifying consistent patterns in recent time.

In network terms, we extract two networks for each of the three years in 2013–2015:

1. A publication network, *G*_*p*,*t*_. This is a weighted graph in which an edge counts the number of times that researchers *i* and *j* were coauthors on one published article in year *t* (we indicate the edge weight as $g_{{i,j}_{p,t}}\geq 1$);
2. A grant network, *G*_*g*,*t*_. This is a weighted graph in which an edge counts the number of times *i* and *j* were PIs or Co-PIs on the same awarded grant in year *t* (the relative edge weight is $g_{{i,j}_{g,t}}\geq 1$).

The *union* network, *G*_*u*,*t*_, is obtained by merging *G*_*p*,*t*_ and *G*_*g*,*t*_. This cross-sectional network includes all nodes in the publication and grant network, with the union edge weight being $g_{{i,j}_{u,t}}=g_{{i,j}_{p,t}}+g_{{i,j}_{g,t}}$. *G*_*u*,*t*_ is a weighted network where individuals *i* and *j* are connected (i.e., $g_{{i,j}_{u,t}}\geq 1$) if they were co-authors on at least one publication or co-investigators on at least one grant in year *t*. The cross-sectional union network is the most comprehensive representation of scientific collaborations at UF that can be obtained from our data for a given year *t*. *G*_*u*,*t*_ is cross-sectional in that it is observed at a single point in time (year *t*). An overall representation of all publication and grant collaborations over 2013–2015 is obtained by creating an overall union network, *G*_*u*_, as the sum of the three union networks in 2013–2015: *G*_*u*_ = *G*_*u*,2013_ + *G*_*u*,2014_ + *G*_*u*,2015_. Isolates (i.e. investigators with no collaborations on publications or grants) are excluded from all publication and grant networks analyzed in this paper.

A concern with this type of data might be that three years are not a sufficiently long span of time to capture the dynamics and time variation of collaborations, for two main reasons. In the first place, the set of co-authors in publications might remain constant over that period of time. Secondly, budget periods of awarded grants might last longer than this time frame, consequently a time window of three years might register only the activation of collaborations, but not the dissolution. While the solution to the former issue heavily relies on the structure of the data (e.g. whether there is a variation in co-authorships or not), the latter issue may be addressed considering all collaborations that are active during 2013–2015, not only the ones that started within this time frame. In this way, we can observe the dissolution of existing collaborations for grants that started before 2013 and ended before 2015, and the activation of new collaborations for grants that started after 2013. Appendix A in S1 File provides evidence that this approach allows our three-year data to capture the dynamic process of collaborations’ activations and dissolutions.

The main characteristics of the union networks are displayed in Table 1. Although the overall number of researchers involved in scientific collaboration at UF decreases over 2013–2015, the main structural characteristics of the networks remain constant in time. Most interactions occur within the main (or giant) connected component, which is the largest set of nodes that are directly or indirectly connected. This suggests that most departments are connected to each other via direct or indirect paths that comprise at least an interdisciplinary, cross-departmental collaboration. While UF has one very large main component, other universities may not have one due to separation of campuses, such as medical campuses in separate locations. Density (the number of existing ties as a proportion of all possible ties) and modularity (a measure of the extent to which the network is divided into separate communities) are approximately constant in the three years, indicating that the overall level of collaborations and their community structure are persistent over time.

**Table 1:** Structural characteristics of union networks (*G*_*u*,*t*_).

	*G*_*u*,2013_	*G*_*u*,2014_	*G*_*u*,2015_	*G*_*u*_
Number of nodes	4414	4038	3358	6414
Number of edges	11950	9783	8363	2.154
Density	0.0012	0.0012	0.0015	0.0010
Modularity	0.8411	0.8558	0.8466	0.7777
Number of components	237	264	260	220
% of nodes in the giant component	85.09	81.08	77.43	90.80

## 3. Methods

The rich set of interactions captured in our data entails two major challenges. First, the data include a large amount of interactions over time. Analyses of such large network data normally require extremely computer-intensive calculations. Second, there is a constant variation of these interactions over time (see Appendix A in S1 File), which is a possible source of confounding factors: sporadic and temporary instances of collaboration may generate a noise that can bias our comprehension of systematic collaboration patterns. We address these issues by developing a method to aggregate and summarize data from multiple years, reducing the computational burden of network analyses, while also accounting for a significant portion of the variation in interactions between years.

Our aim is to identify persistent patterns of collaboration over time. Research on scientific collaboration networks has showed that previous collaborations affect the choice of future collaborators. On the one hand, investigators may find it easier to collaborate with someone with whom they have already worked (for example, because the time to find this collaborator and the coordination efforts are minimized). On the other hand, investigators show a propensity to collaborate with colleagues who have become influential because of a large number of other previous collaborations, in a pattern of preferential attachment [17,41]. Similar research interests and network proximity also increase the propensity of collaboration between two investigators [39]. Thus, researchers who are interested in the same topics are more likely to directly work together or to share a high number of collaborators over time. The result is the emergence of a community network structure [46–47], in which subgroups of nodes are densely connected with each other, and have a high number of adjacent nodes in common (high internal clustering), while being more sparsely connected with the rest of the network.

We analyze the evolution of this community structure by developing a two-step strategy to identify *persistent* research communities. First, we detect clusters that signal the presence of collaborative subgroups in yearly cross-sectional networks (*G*_*u*,2013_, *G*_*u*,2014_, *G*_*u*,2015_). Second, we identify sets of researchers who have consistently been part of the same collaborative subgroups, filtering out temporary collaborations that were only in place for a limited time.

### 3.1. Detecting inter-temporal research communities

In the first step we extract collaborative subgroups in each of the three cross-sectional union networks (*G*_*u*,2013_, *G*_*u*,2014_, *G*_*u*,2015_) using the Louvain community detection algorithm [48]. This algorithm was specifically designed for large-scale networks with thousands of nodes, similar to the ones in our data, and has been reliably used in several applications [35]. The method optimizes the network modularity associated with a given partition into subgroups. Intuitively, modularity quantifies the extent to which subgroups or clusters of nodes are internally well-connected, and are sparsely connected with the rest of the network [49]. The Louvain method analyzes investigators’ interactions both at a bilateral and multilateral level, and identifies the most efficient partition of the network structure into subgroups. The algorithm begins by assigning each node (investigator) to its own subgroup, thus starting with as many subgroups as there are nodes. Then, all nodes are assigned to a reduced set of subgroups that increases modularity as much as possible, determining the fraction of edges falling *within* subgroups to be higher than the fraction of edges existing *across* subgroups. The resulting subgroups are used to generate a reduced graph, in which each node represents a subgroup, and edges measure the connections between the subgroups in the original network. The algorithm iteration is repeated on the reduced graph. In this way, each iteration generates smaller and smaller reduced graphs, and the algorithm ends when modularity can no longer be increased. Applied to our networks, the Louvain algorithm returns a partition of investigators into collaborative subgroups within the network of each year *t*. In this partition, each investigator *i* is associated with a collaborative subgroup $S_{k_{t}}$, where *k*_*t*_ *ϵ* {1,…,*n*_*t*_}, and *n*_*t*_ is the total number of collaborative subgroups detected at time *t*. Thus, the Louvain algorithm yields three partitions of investigators into yearly collaborative subgroups, one for each year in 2013–2015.

The three partitions are used to construct co-membership networks, in which the tie between two researchers indicates that they were members of the same collaborative subgroup $S_{k_{t}}$ for more than one year. Different types and degrees of subgroup co-membership are possible. We construct two types of co-membership networks, namely a cross-sectional and an inter-temporal type. In cross-sectional co-membership networks, individuals *i* and *j* are connected if they were co-members of the same yearly collaborative subgroup $S_{k_{t}}$ for either three or two years. The two years do not need to be consecutive. This co-membership network is a weighted graph, *G*_*c*_, whose edge weight $g_{{i,j}_{c}}$ is the number of years that *i* and *j* were in the same community ($0\leq g_{{i,j}_{c}}\leq 3$). Two binary cross-sectional co-membership networks are generated by setting two different thresholds on $g_{{i,j}_{c}}$: *G*_*c*,1_ includes all edges whose weight is $g_{{i,j}_{c}}\geq 2$ (two investigators are linked if they were in the same collaborative subgroup for *at least* two, not necessarily consecutive, years out of three); co-membership network *G*_*c*,2_ only includes edges whose weight is $g_{{i,j}_{c}}=3$ (two investigators are linked if they were in the same collaborative subgroup for all the three years). Table 2 summarizes the co-membership notation.

**Table 2:** Notation summary.

Approach	Graph	Edge weight
Cross-sectional	*G*_*c*,1_	$g_{{i,j}_{c,1}}=I(g_{{i,j}_{c}})≔\left\{ \begin{array}{c} 1\;if\;g_{{i,j}_{c}}\geq 2\\ 0\;otherwise \end{array}\right.$
	*G*_*c*,2_	$g_{{i,j}_{c,2}}=I(g_{{i,j}_{c}})≔\left\{ \begin{array}{c} 1\;if\;g_{{i,j}_{c}}=3\\ 0\;otherwise \end{array}\right.$
Inter-temporal	*G*_*y*,1_	$g_{{i,j}_{y,1}}=I(g_{{i,j}_{y}})≔\left\{ \begin{array}{c} 1\;if\;g_{{i,j}_{y}}\geq 1\\ 0\;otherwise \end{array}\right.$
	*G*_*y*,2_	$g_{{i,j}_{y,2}}=I(g_{{i,j}_{y}})≔\left\{ \begin{array}{c} 1\;if\;g_{{i,j}_{y}}=2\\ 0\;otherwise \end{array}\right.$

In the inter-temporal co-membership networks, two investigators are connected if they are part of the same *inter-temporal* collaborative subgroup over multiple years. An inter-temporal subgroup is defined as a pair of collaborative subgroups from consecutive years, *S*_*k*,*t*_ and *S*_*k*,*t*+1_ (*tϵ*{2013,2014}), that maximize the following overlap measure [41]:

$$\phi =\frac{N(S_{k,t})\cap N(S_{k,t+1})}{N(S_{k,t})\cup N(S_{k,t+1})}$$

**Fig 1:**
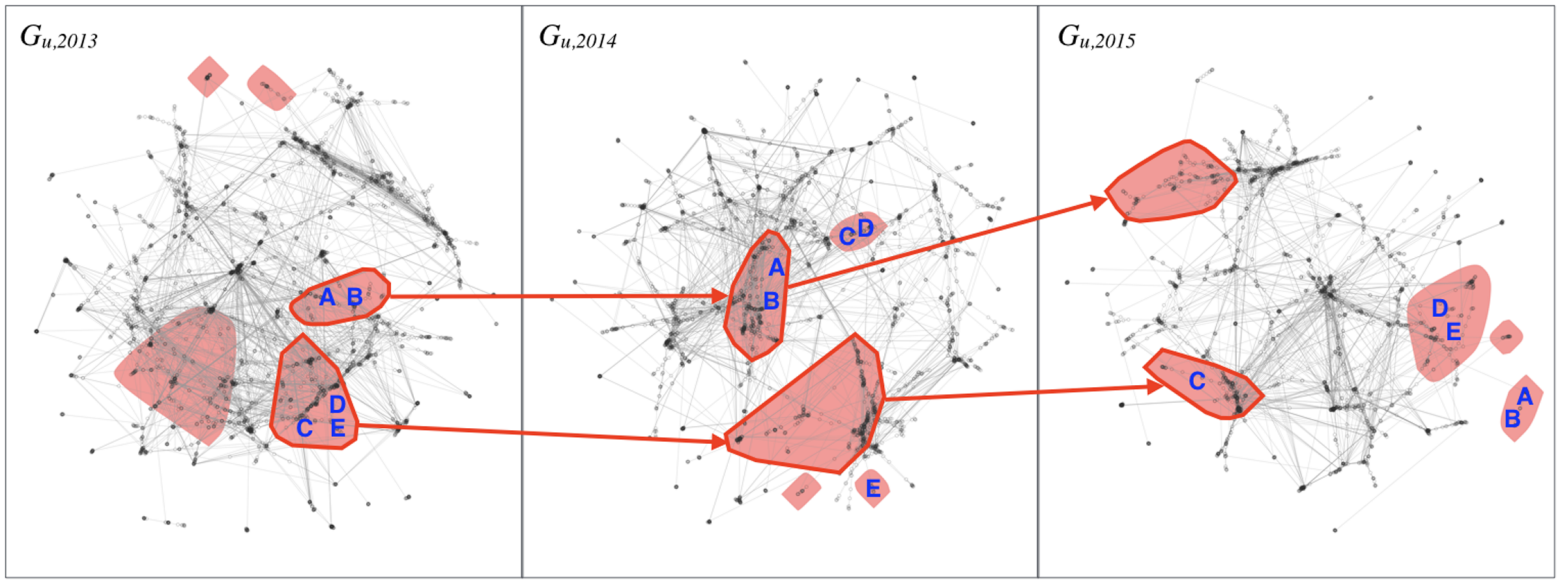
Extracting co-membership relationships from collaborative subgroups. Red shaded areas are *yearly* collaborative subgroups. Red arrows indicate sequences identified as *inter-temporal* collaborative subgroups. Investigators A and B are in the same yearly collaborative subgroup for all the 3 years: *g*_*c*_(*A*,*B*) = 3. However, only for 2 years are they in the same inter-temporal collaborative subgroup: *g*_*y*_(*A*,*B*) = 1. Investigators C and D are in the same yearly collaborative subgroup for two consecutive years (*g*_*c*_(*C*,*D*) = 2), but this is not an inter-temporal subgroup (*g*_*y*_(*C*,*D*) = 0). Investigators D and E are in the same yearly collaborative subgroup for two non-consecutive years (*g*_*c*_(*D*,*E*) = 2), but they are never in the same inter-temporal collaborative subgroup (*g*_*y*_(*D*,*E*) = 0).

Where *N*(∙) is the set of all nodes in community *S*_*k*_. This overlap measure (also known as the Jaccard index of similarity between two sets) is maximized by a pair of collaborative subgroups A and B when subgroup *A* in year *t* shares the highest proportion of nodes with subgroup *B* in year *t+1*, indicating that the core of investigators who were in *A* in *t* are also in *B* in *t+1*; or, in other words, that *A* and *B* are fundamentally the same collaborative subgroup over two years. In the inter-temporal co-membership network, *G*_*y*_, the edge weight $g_{{i,j}_{y}}$ counts the number of times that *i* and *j* have been members of an *inter-temporal* collaborative subgroup (*S*_*k*,*t*_,*S*_*k*,*t*+1_). Two binary inter-temporal co-membership networks are constructed, corresponding to two different thresholds on $g_{{i,j}_{y}}$: *G*_*y*,1_ includes all edges for which $g_{{i,j}_{y}}\geq 1$ (two investigators are linked if they have been in the same inter-temporal subgroup for *at least* two consecutive years out of three); *G*_*y*,2_, which only includes edges for which $g_{{i,j}_{y}}=2$ (two investigators are linked if they have been in the same inter-temporal subgroup for three consecutive years). Fig 1 illustrates the concepts of cross-sectional and inter-temporal co-membership.

Our method results in the construction of four different co-membership networks. A co-membership tie between two scientists in these networks indicates that they have tended to be part of the same collaborative subgroups over the three years. This may reveal different types of relationships or similarities between two researchers. Two individuals may be connected in the co-membership networks because they have consistently worked together. They may also be connected if, even without ever working together, they have consistently shared the same collaborators over the years. Thus, a co-membership tie shows that two scientists have similar substantive or methodological research interests, or that they have developed complementary sets of skills and expertise that are sought by the same other collaborators. Scientists who are linked in the co-membership networks have been part of the same work circles and environments, encountering similar ideas and information in their workplace over the years. Therefore, cohesive clusters in co-membership networks can be viewed as research communities of scientists who have consistently shared similar research interests, methods, and scientific approaches to problems. We identify such research communities by applying the Louvain community-detection algorithm again, this time to the co-membership networks. This yields four sets of research communities *C*_*k*,*G*_, one for each co-membership network, where *kϵ*{1,…,*n*_*G*_} and n_*G*_ is the total number of Louvain communities detected in co-membership network G.

Our method is based on a shift from the original collaboration networks, in which a tie indicates direct collaboration between two individuals, to networks of co-membership in collaborative subgroups, in which a tie indicates that two individuals have consistently participated in the same research groups and work circles over time. The co-membership networks aggregate and summarize the information contained in the longitudinal collaboration networks, with the goal of filtering out the noise of transient and intermittent direct collaborations, and extracting the signal of recurrent co-participation in the same scientific circuits over time. Cohesive subgroups detected in co-membership networks are less affected by temporary and random direct collaborations, and reveal groups of researchers who consistently share projects, collaborators, ideas and approaches over time, which we call research communities.

### 3.2. Networks and attributes of research communities

While research communities are only identified on the basis of recurrent interactions over time, with no reference to investigators’ institutional affiliations such as department or college, most communities are likely to mainly include scientists who belong to the same (or close) colleges, departments, institutes or centers. This is the case because common institutional affiliation and discipline obviously facilitate and incentivize working together in a recurrent fashion. At the same time, although most collaborations occur within the identified research communities, some collaborations also exist between investigators who belong to different communities. By construction, these cross-community collaborations are the links that connect researchers from different scientific circuits, cut across well-established groups, and span diverse work environments. Therefore, such collaborations are likely to sustain some of the most innovative, cutting-edge and interdisciplinary projects at a research university. We map these cross-community collaborations by constructing community-level networks in which nodes represent research communities and edges represent the density of collaborations between two different communities. We compute the density of collaborations between two communities, *C*_*f*_ and *C*_*k*_, as follows (note that to avoid heavy notation, the G term referring to the co-membership network will not be written explicitly)

$$D_{C_{f}C_{k}}=\frac{e(C_{f},C_{k})}{\sqrt{N(C_{f})N(C_{k})}}$$

Where *C*_*f*_ and *C*_*k*_ are two different communities detected in the co-membership network *G* (one of the four co-membership networks), and *e*(*C*_*f*_,*C*_*k*_) is the number of edges in *G*_*u*_ (the three-year overall union network for 2013–2015) involving a member of *C*_*f*_ and a member of *C*_*k*_. $D_{C_{f}C_{k}}$ is a measure of density of inter-group connections adjusted by group size [50]. In our case, $D_{C_{f}C_{k}}$ measures the density of publication and grant collaborations that occurred over the whole three-year period between members of two different communities. For each co-membership network *G*, we obtain a weighted network of cross-community collaborations, in which nodes are the research communities extracted from *G*, and $D_{C_{f}C_{k}}$ is the weight of the edge between any two communities *C*_*f*_ and *C*_*k*_. This weighted network is then converted to a binary network, *T*_*G*_, by keeping an edge between communities *C*_*f*,*G*_ and *C*_*k*,*G*_ if $D_{C_{f}C_{k}}$ is greater than the median of its distribution among all pairs of communities extracted from *G*. In other words, *T*_*G*_ is a network in which two communities are connected if the density of collaborations between them is greater than the median density of collaboration between any two communities from the same co-membership network. In this way, we ensure that the architecture of these networks is not biased by contextual effects (e.g., occasional collaborations).

Two additional networks are created to represent spatial proximities between research communities:

- *B*_*G*_ is a network of *building* proximities between communities. We measure building proximity between *C*_*f*_ and *C*_*k*_ as the number of pairs of investigators {*i*,*j*} such that *i* belongs to community *C*_*f*_, *j* belongs to community *C*_*k*_, and *i*’s and *j*’s offices are located in the same *building*. *B*_*G*_ is a weighted network in which each node is a research community and the edge weight is the building proximity measure.
- Similarly, *F*_*G*_ is a network of *floor* proximities between communities. Floor proximity between *C*_*f*_ and *C*_*k*_ is measured as the number of pairs of individuals {*i*,*j*} such that *i* belongs to *C*_*f*_, *j* belongs to *C*_*k*_, and *i*’s and *j*’s offices are located in the same *floor* of the same building. In *F*_*G*_ each node is a research community and the edge weight is the floor proximity measure.

In the network formation models, cross-community collaborations are the dependent variable, which is explained by community attributes and network structural factors. We obtain a number of attributes that describe the research profile of each community. The starting hypothesis is that the determinants of persistent collaborations between communities include similarity of expertise (i.e., scientists tend to collaborate when they have similar backgrounds and skills) and complementarity of expertise (i.e., scientists tend to collaborate when they have complementary, non-overlapping backgrounds and skills) [21,43]. These concepts are operationalized measuring the distribution of disciplinary backgrounds of a community’s members, as represented by members’ departments and colleges. To capture expertise similarity, we calculate the modal value of the distribution, i.e., the most common department or college among a community’s members. To quantify expertise complementarity, we obtain the generalized variance of the distribution, which can be interpreted as the probability that two randomly extracted researchers in the community share the same department or college [51].

To analyze the effect of the UF CTSI on cross-community collaborations, we also measure the use of CTSI research services by members of a community. For each community, we calculate the proportion of its members who have used CTSI services in 2013–2015. In each or the four sets of communities, 33% is approximately the 75th percentile of the distribution of this proportion–in other words, in 75% of all communities less than one third of the members have used CTSI services. We create a community-level dummy variable for CTSI service use, whose value is 1 if 33% or more of the community members have used CTSI services in 2013–2015, and 0 otherwise.

Finally, we calculate the following network metrics for each community in the overall union network *G*_*u*_:

- Density of collaborations within the community;
- Bridging centrality. This index measures the extent to which community’s members function as a bridge between unconnected areas of the UF collaboration network. This is a group version of the bridging centrality measure by [52]. For each community *C*_*f*_, we select the set *e*(*C*_*f*_,*G*_*u*_ − *C*_*f*_) of all edges connecting *C*_*f*_ to the rest of the network *G*_*u*_. Then, we calculate the average decrease in overall network cohesion (measured as inverse average path length in *G*_*u*_,) caused by the removal of edges in *e*(*C*_*f*_,*G*_*u*_ − *C*_*f*_). A community *C*_*f*_ has higher bridging centrality if the removal of the edges connecting it to the rest of the network causes a higher decrease in overall network cohesion. In other words, higher bridging values are associated to those communities that are more critical in reducing path lengths between all investigators in *G*_*u*_.

### 3.3. ERG models for collaborations between research communities

We use ERG models [53] to analyze the formation of cross-community collaborations, that is, the formation of links in the four cross-community collaboration networks (*T*_*G*_). Similar to a logistic model, an ERGM is used to estimate the effect of a set of explanatory variables (e.g. discipline similarity) on the probability of an event, the event being in this case the existence of a link (i.e., above-median density of collaborations) between two research communities. ERGM parameter estimates, for example, can indicate the extent to which the probability of a link between communities *C*_*f*_ and *C*_*k*_ increases when *C*_*f*_ and *C*_*k*_ share the same modal value of department affiliation among their members. Furthermore, a crucial feature of ERGMs is the ability to model the generation of networks based on both exogenous and endogenous factors, controlling not only for dyadic effects (e.g. similarity), but also for more complex structural patterns such as transitivity and star structures. In the next section, we present ERG estimates for the impact of community attributes and network factors on the formation of each cross-community network *T*_*G*_.

## 4. Results

Table 3 summarizes the structural characteristics of collaborative subgroups obtained for each cross-sectional union network (*G*_*u*,2013_,*G*_*u*,2014_,*G*_*u*,2015_). For comparison purposes, we also include statistics for collaborative subgroups obtained from the overall union network (***G***_***u***_). The full cumulative distribution functions are displayed in Appendix B in S1 File. Except for a slight decrease in average subgroup size, the characteristics of cross-sectional networks appear to be mostly constant over time. Additional evidence is provided in Appendix B in S1 File (Figure B in S1 File) by plots of the cumulative distribution functions for each structural characteristic in Table 3. The significant overlap of the distributions from different yearly networks indicates that characteristics of collaborative subgroups tend to remain the same in 2013–2015. Note that this is the case even though most collaborations do change over time, with old collaborations ending and new ones starting (see Appendix A in S1 File).

**Table 3:** Main characteristics of collaborative subgroups in cross-sectional union networks.

	*G*_*u*,2013_	*G*_*u*,2014_	*G*_*u*,2015_	*G*_*u*_
	Mean (SD)	Mean (SD)	Mean (SD)	Mean (SD)
Number of nodes (subgroup size)	15.76 (43.16)	13.19 (35.13)	11.26 (28.84)	25.05 (79.27)
Average degree in subgroup	4.74 (4.65)	4.31 (4.09)	4.40 (4.31)	1.96 (1.75)
Subgroup density	0.78 (0.33)	0.78 (0.32)	0.80 (0.31)	0.80 (0.32)
Subgroup diameter	2.38 (2.89)	2.32 (2.77)	2.15 (2.49)	2.32 (2.95)

Most researchers work in relatively small groups of 10–15 colleagues, with a high number of internal collaborations, as suggested by the high density (0.78–0.80) and low network diameter (2.1–2.3). Despite the variance in subgroup size, the number of collaborations per investigator (average degree) remains relatively constant, with the average scientist working with 4–5 colleagues in the same subgroup.

**Fig 2:**
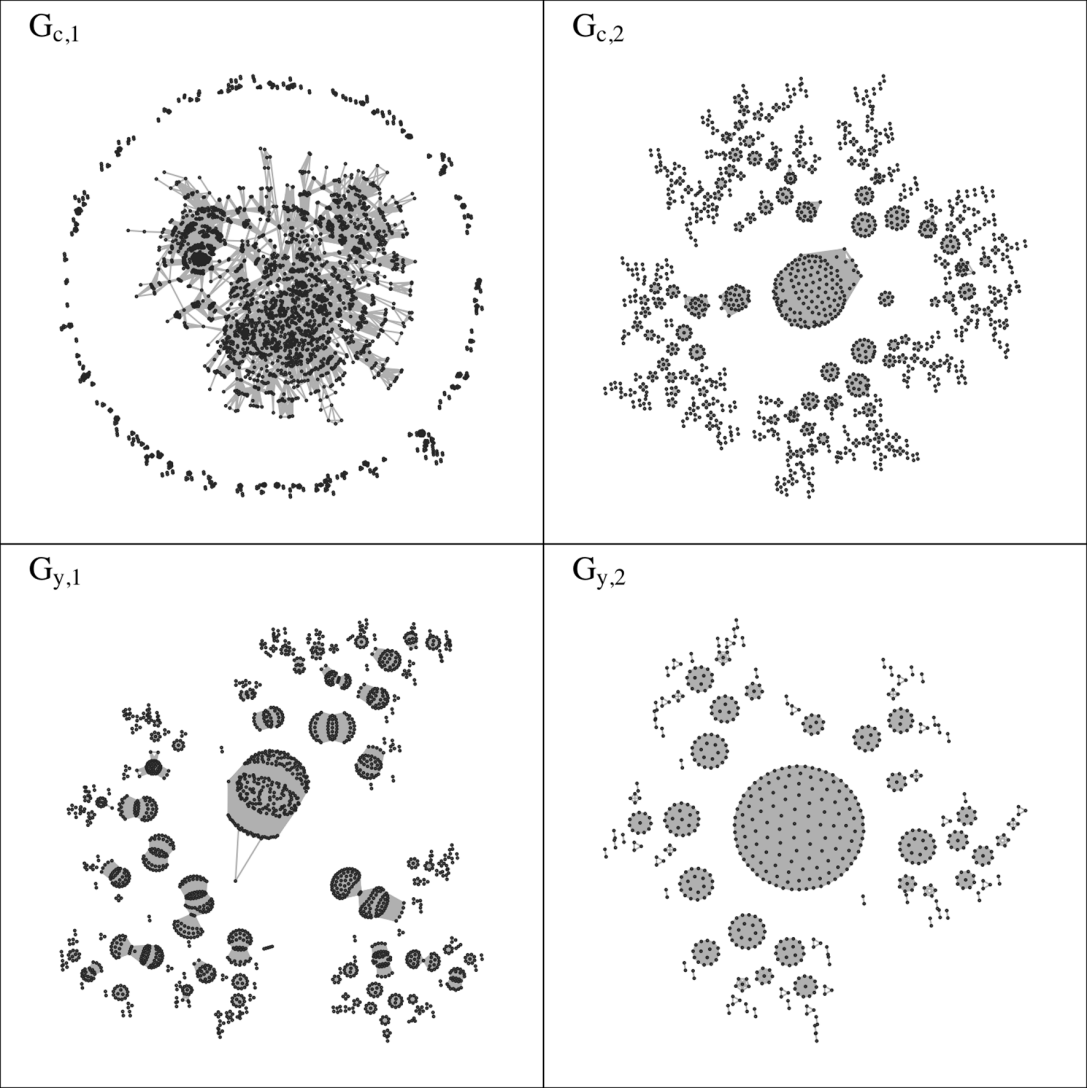
Cross-sectional and inter-temporal co-membership networks.

The networks of co-membership in collaborative subgroups are visualized in Fig 2, with some of their structural characteristics displayed in Table 4. Different definitions of co-membership appear to determine different structural patterns in these networks. The cross-sectional co-membership networks result from a weaker (more inclusive) definition of co-membership, such that two investigators are connected by co-membership if they were in the same yearly collaborative subgroup for two (not necessarily consecutive) years or three years, but not necessarily in the same *inter-temporal* collaborative subgroup. Thus, two researchers might share a *different* collaborative subgroup each year, and still be connected by cross-sectional co-membership. By contrast, a stronger (stricter) definition of co-membership is applied in the inter-temporal networks, in which two investigators are only connected if they were co-members of the same *inter-temporal* subgroup, sharing only one circle of colleagues for two or three *consecutive* years.

**Table 4:** Structural characteristics of co-membership networks.

	Cross-sectional co-membership networks		Inter-temporal co-membership networks	
	*G*_*c*,1_	*G*_*c*,2_	*G*_*y*,1_	*G*_*y*,2_
Number of nodes	2919	1219	1672	535
Number of edges	11543	4186	5831	1627
Density	0.0027	0.0056	0.0042	0.0114
Modularity	-0.0006	0.0020	0.7654	0.7828
Number of components	37	21	44	24
% of nodes in the giant component	95	94	89	82

The size of the giant component, which is a measure of overall network connectedness, is one of the structural characteristics affected by the definition of co-membership. In the cross-sectional co-membership networks, it is easier for two investigators to be co-members of the same collaborative subgroup, which results in higher overall connectedness and larger giant components (as a proportion of the whole co-membership network). By contrast, inter-temporal co-membership networks are characterized by lower connectedness and smaller giant components. Secondly, weaker definitions of co-membership are also associated to a lower number of isolates (nodes with no connections) and consequently larger network size (because isolates are removed from the co-membership networks). This is the case because, by cross-sectional definitions of co-membership, investigators are more likely to be co-members of the same subgroups with other colleagues, and therefore establish co-membership ties and not be isolates. This is reflected in the larger size of cross-sectional co-membership networks compared to inter-temporal co-membership networks.

Finally, the value of modularity also changes between more inclusive and stricter co-membership networks. In the inter-temporal co-membership networks investigators coalesce into closer subgroups representing the small circles of colleagues that they have constantly shared over the years. This generates a co-membership graph with highly distinct, clear-cut and insular communities, as indicated by the high modularity. By contrast, in cross-sectional co-membership networks some investigators are able to maintain co-membership ties with a wider variety of colleagues, emerging as bridges between different scientific circles. The result is a less clear-cut division into cohesive subgroups, and correspondingly lower modularity values. The thresholds (numbers of years) applied in the co-membership definitions seem to affect the structure of co-membership networks as well. Networks *G*_*c*,2_ and *G*_*y*,2_ are very similar even though they apply different co-membership definitions. This is because in both networks investigators are only connected if they have been in the same collaborative subgroup for all the years 2013–2015 (although that must be the same, inter-temporal collaborative subgroup for a connection to exist in *G*_*y*,2_).

Table 5 shows means and standard deviations of community attributes used in the ERG models (see Appendix B in S1 File for the full cumulative distribution functions). Research communities display similar characteristics in each of the four sets. The generalized variance of department affiliation (0.51–0.58) indicates a significant level of department diversity in these communities, with more than 50% probability that two randomly extracted community members belong to different departments. As expected, college diversity is lower (0.31–0.37), suggesting that most research communities gather investigators from one or very few colleges. Because colleges often represent broad disciplinary areas, this is consistent with the idea that scientific collaboration is facilitated by discipline similarity. In other words, two investigators from the Department of Neurology and the Department of Neuroscience (both in the College of Medicine) are more likely to be in the same research community, rather than two investigators from the College of Medicine and the College of Engineering. Communities share a similar density of interactions. This shared feature most likely occurs because most of the researchers have few single-year collaborations, as reported in Table 3. Therefore, all methods capture similar overall levels of interaction, even though they apply different restriction criteria to identify persistent collaborations.

**Table 5:** Attributes of research communities.

	Cross-sectional co-membership communities		Inter-temporal co-membership communities	
	Mean (SD)		Mean (SD)	
	$T_{G_{c,1}}$	$T_{G_{c,2}}$	$T_{G_{y,1}}$	$T_{G_{y,2}}$
Size	23.16 (52.70)	6.44 (10.25)	15.33 (29.14)	10.91 (18.62)
Generalized variance of department affiliation	0.50 (0.3099)	0.5486 (0.23)	0.58 (0.26)	0.5575 (0.28)
Generalized variance of college affiliation	0.33 (0.28)	0.31 (0.26)	0.37 (0.23)	0.34 (0.26)
Within-community density	0.60 (0.32)	0.67 (0.24)	0.64 (0.27)	0.58 (0.30)
Bridging	1.44 (3.64)	1.51 (3.80)	0.31 (0.60)	1.31 (3.56)
N	189	126	109	49

**Fig 3:**
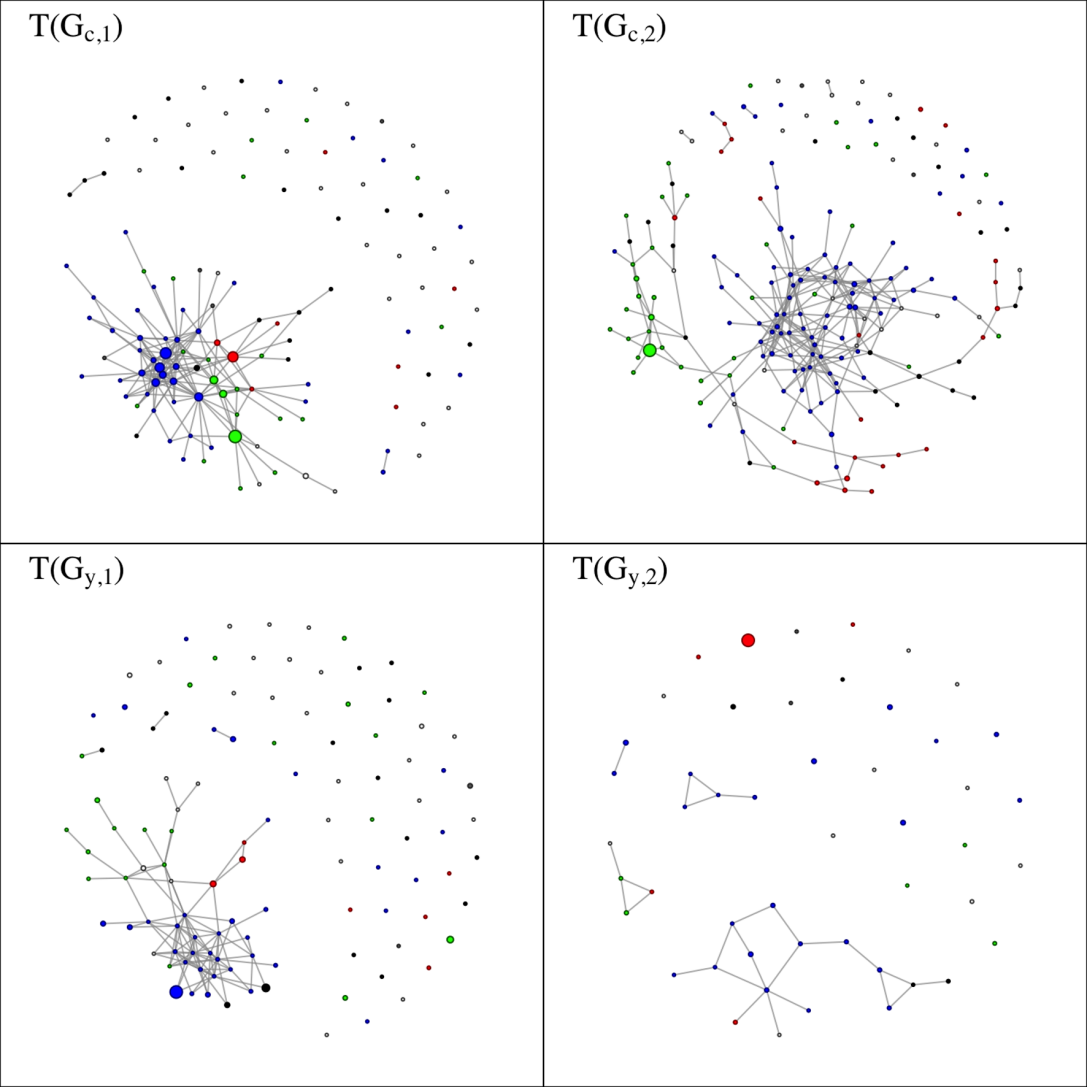
Networks of collaborations between research communities. Each node is a community, an edge represents above-median density of collaborations between communities ($D_{Z_{f}Z_{k}}$). Node size represents the number of investigators in the community. Node colors represents the modal disciplinary area of investigators in the community (Blue = Health sciences, Red = Engineering, Green = Agricultural and Food Sciences, Black = College of Liberal Arts and Sciences, White = Other).

The structures of the four cross-community collaboration networks show a higher degree of variation (Table 6 and Fig 3; also see Figure C in S1 File). Such variation is mostly explained by the different definitions of co-membership from which the research communities result, including the weaker, more inclusive cross-sectional definition and the stronger, more restrictive inter-temporal definition. These co-membership definitions imply different approaches for filtering out temporary collaborations and identify persistent professional interactions. Consistently with Table 4, community networks resulting from cross-sectional co-membership include a higher number of nodes (i.e., research communities). Furthermore, the weaker cross-sectional definition of co-membership identifies more permeable and open communities, whose members are more likely to maintain ties with scientists from other communities as well. As a result, $T_{G_{c,1}}$ and $T_{G_{c,2}}$ are characterized by a higher number of cross-community collaborations, reflected in higher average degree. In contrast, $T_{G_{y,1}}$ and $T_{G_{y,2}}$ are sparser and less cohesive, with fewer cross-community collaborations, fewer communities in the main component, and a higher proportion (about 50%) of isolated communities. It should be kept in mind that an isolated research community is one with no collaborations, or lower-than-median densities of collaborations with other communities. It is an expected finding that all the four community networks include high numbers of isolates, particularly when they are generated by stricter definitions of co-membership. By construction, communities are cohesive groups of investigators who tend to work mostly with each other, and more rarely with other communities. Thus, an isolated research community should be considered the norm in the networks visualized in Fig 3. The significant portion of isolated communities indicates, as expected, that most higher-than-median densities of cross-community collaborations are clustered among a limited number of research communities, which are very open to the outside.

**Table 6:** Structural characteristics of cross-community collaboration networks.

	Cross-sectional co-membership networks		Inter-temporal co-membership networks	
	$T_{G_{c,1}}$	$T_{G_{c,2}}$	$T_{G_{y,1}}$	$T_{G_{y,2}}$
Number of nodes	189	126	109	49
Number of edges	580	356	230	52
Average degree (standard deviation)	9.20 (8.89)	8.47 (13.58)	6.3303 (10.66)	3.18 (3.98)
Density	0.0016	0.0022	0.0002	0.0022
Modularity	0.60	0.39	0.39	0.66
Number of components	36	54	61	28
% of nodes in the giant component	78	55	42	30
% of isolates	16	40	52	48

### 4.1. Network formation analysis

While a high number of research communities maintain lower densities of collaborations with the outside, all networks in Fig 3 do exhibit a significant amount of cross-community collaborations with higher-than-median densities. These inter-community ties can be regarded as the vehicles for the most interdisciplinary, and likely innovative, collaborations, which occur between separate and diverse scientific circles. Table 7 reports results of an ERGM analysis of the formation of inter-community ties. For each *T*_*G*_ network, a curved ERGM is used to assess the impact of a set of factors in shaping the likelihood of connection between two different research communities. ERGM estimates are in log odds, thus the probability of a connection is obtained as $\frac{e^{-x}}{1+e^{-x}}$, where *x* is the estimated coefficient of a given covariate.

**Table 7:** ERGM results.

	Dep. Var.: Link in network *T*_*G*_			
	Cross-sectional co-membership networks		Inter-temporal co-membership networks	
	$T_{G_{c,1}}$	$T_{G_{c,2}}$	$T_{G_{y,1}}$	$T_{G_{y,2}}$
Edges	-4.7159*** (0.1818)	-3.9486*** (0.2767)	-3.6029*** (0.3664)	-4.1178*** (0.7375)
**GwEsp**	0.9149*** (0.0838)	0.405*** (0.0792)	0.6587*** (0.1328)	0.7287** (0.4311)
Building (*B*_*G*_)	-0.0093 (0.0175)	0.0093** (0.0042)	0.0058 (0.006)	0.056** (0.0275)
Floor (*F*_*G*_)	0.1634*** (0.0384)	0.0026 (0.0092)	0.0431** (0.0181)	0.0237 (0.0606)
**Same department: mode value**	1.4463*** (0.2336)	1.0095*** (0.3713)	1.7873*** (0.631)	1.7389** (0.9691)
**Same college: mode value**	0.9069*** (0.1253)	1.1548*** (0.1701)	0.6787*** (0.2015)	0.7748** (0.469)
Department variance: difference	-0.3728 (0.2888)	-0.0842 (0.391)	-1.3272** (0.6656)	-1.0146 (1.1975)
College variance: difference	-0.4484 (0.2767)	-0.2754 (0.4127)	-0.2292 (0.551)	0.959 (1.1583)
Density: difference	-0.3017 (0.3076)	-1.2022*** (0.3612)	-0.7617 (0.5236)	-0.2674 (1.0909)
Bridging measure: difference	0.0047 (0.0116)	-0.0309 (0.0322)	-0.0733 (0.0733)	-0.4779 (0.543)
Number of nodes: difference	-0.0025 (0.0049)	0.0057*** (0.0012)	-0.0044** (0.002)	-0.0354 (0.0373)
CTSI: both low	-0.2105 (0.1359)	-0.0236 (0.1917)	-0.0224 (0.3055)	0.5112 (0.531)
**CTSI: both High**	0.4244*** (0.1355)	0.463** (0.2451)	0.511** (0.2235)	1.5108*** (0.5492)
Isolates	0.0917 (0.2595)	1.3767*** (0.3309)	1.2412*** (0.3908)	0.4748 (0.5457)
AIC	2433.3995	1101.6016	685.0428	216.6608
N. Obs.	189	126	109	49

Note: ERGM estimated coefficients and standard errors (in parentheses) are reported.

Coefficients are in log–odds.

*, **, *** indicate statistical significance at the 10, 5 and 1 percent levels.

The ERGM estimates return similar findings across the four cross-community collaboration networks. In all networks, three main factors are identified as having a positive and statistically significant effect on the probability of collaboration between different communities: GwEsp (geometric weighted edge-wise partners), department and college affiliation, and use of CTSI research services. The GwEsp parameter captures the effect of transitivity, whereby two communities *C*_*f*_ and *C*_*k*_ have a higher likelihood of collaboration if they tend to work with the same other communities. GwEsp controls for transitivity, while accounting for diminishing marginal effects of additional shared collaborators. In other words, GwEsp measures transitivity while accounting for the fact that there is a limit to the number of possible shared collaborators that two communities can maintain (both for reason of time and for the low number of communities with similar interests).

The statistically significant impact of department and college affiliation on the probability of collaboration is an expected result, confirming that even cross-community collaborations require similar or complementary interests and backgrounds, and perhaps a set of shared research methods. We find that use of CTSI services has a positive and significant effect on cross-community collaborations. In addition, spatial closeness, as measured by building and floor proximity, also has a positive and significant impact on inter-community collaborations. A higher number of connections in the proximity networks *B*_*G*_ or *F*_*G*_ increases the probability of collaboration between different groups. On the other hand, differences in the bridging centrality measure between communities do not effectively predict collaboration. This suggests that a common tendency to engage in inter-disciplinary collaborations (i.e., both nodes having a high bridging centrality) does not necessarily determine a higher likelihood of collaboration between groups. The effect of size difference on the probability of inter-community connection is not consistent across the four models. This could be due to the community size coefficient capturing different effects simultaneously, including the tendency of equally large communities to maintain more collaborations, and the propensity of smaller communities to join larger groups.

Each estimated ERGM can be interpreted as a generative model representing a process that governs local tie formation in each pair of nodes. The baseline is provided by the “Edges” coefficient, a sort of ERGM intercept, indicating the average likelihood of connection between two nodes in the observed network. In our four cross-community networks, two nodes are connected, on average, with probability 0.009, 0.018, 0.026, and 0.016, respectively (the logistic transformations of coefficients in Table 7). In addition, for example, if two nodes also share the same modal value for department affiliation, they have 81%, 73%, 85%, and 85% higher probabilities of connection, respectively. However, it should be noted that the actual collaborations between the two communities do not necessarily involve investigators from the same department; they may involve investigators from any of the departments represented in the communities. The effect is associated to the modal department in the two communities being the same. In other words, if the majority of the members of two communities are from the same department, then the two communities have between 73% and 85% higher probability of displaying a higher-than-median density of collaborations. But these collaborations might take place between any of the members of the two communities, from any department.

**Fig 4:**
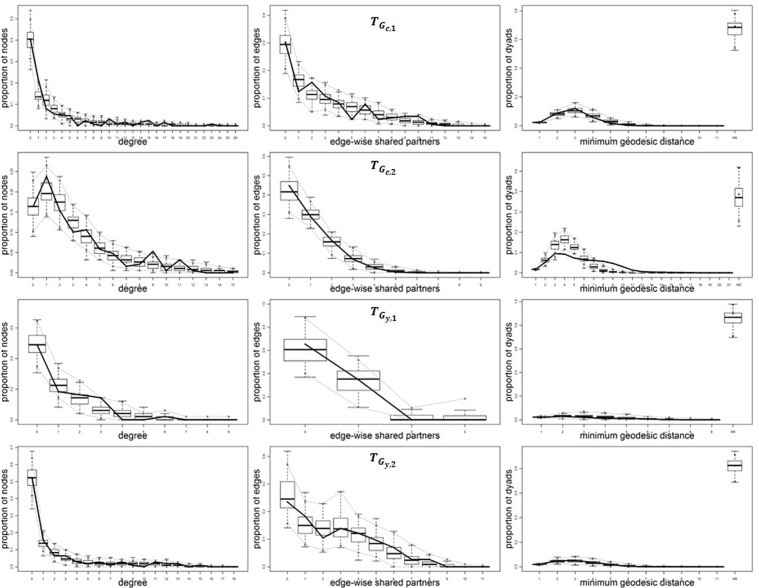
Goodness of fit.

To assess Goodness Of Fit (GOF), these estimates are used to simulate new sets of connections for each sample of nodes, and obtain 100 new instances of each cross-community network. Then, a number of global characteristics of these simulations are measured, including the distributions of degree (i.e., the number of connections of each node), of the number of edgewise shared partners (i.e., the number of partners shared by a linked dyad) [54], and of minimum geodesic distance (i.e., the minimum number of links between two nodes). The distribution of these measures in the observed network is compared with the same distributions in the sample of simulated networks in order to verify if the observed network can be considered as a typical realization from the generative model. This is the case if the average values of the measures in the simulated networks are close to the actual measures in the observed network. These GOF tests assess the extent to which our ERGM specification incorporates all the fundamental drivers of connectivity in the network, and correctly replicates the structural features of the observed network, resulting in unbiased coefficient estimates [55]. The results of the GOF tests on our models are reported in Fig 4. As an example, the top-left figure in the $T_{G_{c,1}}$ panel shows a boxplot of the proportions of nodes (y axis) with each given degree value (x axis) in the networks simulated from the estimated ERGM. Dotted lines indicate the minimum and the maximum values of the boxplots, while the bold line indicates the proportions of nodes observed in the actual inter-community network. We find that in most cases the bold line lies well within the boxplots, showing that, on average, our generative models produce network statistics that are similar to the observed ones, and therefore successfully replicate the actually observed network characteristics. This indicates an overall good fit of our ERG models to the observed inter-community networks.

## 5. Discussion

This paper develops a new method for the identification of different types of long-term scientific collaborations within and between research communities in academic settings, as they emerge from networks of actual collaborations between researchers on publications and grants. We have illustrated two versions on this method. The first, more inclusive and less restrictive version identifies stable research communities based on sets of investigators who have been co-members of the same collaborative subgroups for multiple years. These sets of investigators are allowed to share *different* collaborative subgroups over time, and to be in the same collaborative subgroup for non-consecutive years. The second, stricter version of the method identifies stable research communities on the basis of sets of investigators who have been co-members of essentially the *same* inter-temporal collaborative subgroup for multiple *consecutive* years. The two versions of the method, with two different parameters (thresholds) each, result in four sets of research communities.

We focus on the collaborations between research communities in each set. These are among the most interdisciplinary, cutting-edge, innovative collaborations in a research university because they involve scientists from different academic circles, or clusters of similar disciplinary backgrounds, interests, and approaches. We use ERGMs to identify the main drivers of the generative process of these inter-community collaborations. Some of our findings are expected and consistent with previous research, confirming that similar institutional affiliations, spatial proximity, and network transitivity increase the propensity of collaboration between research communities.

The positive effect of department and college affiliation suggests that homophily plays an important role in scientific networks, with collaborative research usually relying on similar backgrounds and interests among investigators [39]. At the same time, the effect of affiliation indicates that the administrative organization of a university can be both an incentive and a barrier to scientific collaboration. Department meetings, for example, can be a frequent occasion of interaction between colleagues in the same institution. Lack of clear rules on the distribution of indirect costs from extramural awards between different administrative units might discourage grant collaborations between investigators from different colleges. We also find that, at least in the aggregate, overlapping interests between communities (i.e. homophily) have a greater impact than an expertise complementarity on the formation of collaborations, as suggested by the non-significant estimates for department and college generalized variance.

Spatial proximity has a well-known influence on the likelihood of interaction and collaboration [36–38]. In line with this finding, our results show that groups of researchers located in the same floor or building have higher chances to work together. Consequently, one might argue that an appropriate collocation of different departments can mitigate the negative effects of administrative boundaries on interdisciplinary collaborations by creating informal places and occasions of interaction.

The positive effect of network transitivity suggests that scientists tend to introduce their collaborators to one another, a finding which is consistent with a number of previous studies [55–58]. At the same time, network distance is also a proxy for similar backgrounds and interests, as investigators working on the same topics tend to share many collaborators. Thus, the transitivity coefficients might indicate a positive effect of interest similarities on collaborations, above and beyond the similarities captured by common department and college.

Finally, our models find that CTSA services play an important role in increasing the number of interdisciplinary collaborations. All the models consistently indicate that communities with a high number of members who are affiliated with the UF CTSI or use its services have a higher propensity to activate cross-community collaborations. Different mechanisms might be hypothesized to explain this effect. First, common CTSI affiliation might create occasions for informal encounters and interactions in the buildings and offices of the UF CTSI. Second, the use of CTSI services might reveal shared interests in translational science, beyond the interest homophily captured by department and college affiliation (and perhaps by transitivity). Third, CTSI intramural funding opportunities, especially when targeted to interdisciplinary projects, might provide a further incentive for affiliates and service users from different communities to join in common research efforts. These findings, which are consistent with several previous studies [11–15], suggest that research institutes such as the UF CTSI can effectively operate as strategic hubs for diverse groups of scientists located in different areas of the university networks.

## 6. Conclusions

This paper proposes a method to detect research communities in academic settings using longitudinal data on publication co-authorship and co-participation in awarded grants. The same data are used to construct networks of cross-community, interdisciplinary collaborations. The main drivers behind the formation of these networks are identified by ERGMs. We consistently find that interdisciplinary collaborations between research groups are positively affected by homophily and network transitivity; spatial proximity; and the activities of cross-disciplinary academic institutes such as the UF CTSI.

We believe that two points are worth emphasizing about the proposed method. First, this method is particularly useful to summarize data from large-scale, individual-level longitudinal networks. Such networks, including thousands of actors over multiple years, can require extremely high computing power, making statistical analysis unfeasible. Our method provides a way of aggregating individual-level information into more manageable sets of research communities and community-level collaboration networks, which are amenable to both traditional and network-specific statistical analysis.

Second, while we illustrate it with an application to data from the University of Florida and its Clinical and Translational Science Institute, this method can be applied to comparable data from any research university and any academic institute. Such analyses may prove particularly useful to (1) Summarize and describe the research profile of a large university, by identifying its research communities and the interactions among them; (2) Assess the growth of interdisciplinary research at a university, and how that is impacted by specific research policies and services; and (3) Evaluate the role of particular research institutes, such as the UF CTSI, in fostering interdisciplinary collaboration and team science within their institution.

## Supporting information

S1 File (DOCX)
