# Supplementary material for: Detecting and analyzing research communities in longitudinal scientific networks
Source: PLoS One. 2017 Aug 10;12(8):e0182516. doi: 10.1371/journal.pone.0182516 (PMC5552257; doi:10.1371/journal.pone.0182516)

# Appendices

## Appendix 1. Time variation of collaborations in 2013-2015.

This Appendix shows that the 3-year time window considered in this study, 2013-2015, is sufficiently long to encompass time-varying collaborations. A (union) collaboration between researchers *i* and *j*, as defined and analyzed in the paper, exists in year *t* if *i* and *j* either appear as co-authors on one or multiple peer-reviewed articles in *t*, or co-participate as PIs, multiple PIs or co-PIs in one or multiple awarded grants that are active in *t*. Importantly, awarded grants have a *start* and an *end* date, and therefore a duration, unlike articles, which only have a publication date and no duration. Our data comprise all University of Florida extramurally awarded grants that are active in the 2013-2015 time window. These are funded by a wide variety of mechanisms and entities, including federal and state agencies, foundations and societies, local and regional government programs, and corporations and companies. Crucially, the UF grants that are active in 2013-2015, and are included in our data, may have start dates before 2013 or end dates after 2015; they may also have start or end dates within the 2013-2015 window. These grants have a variety of durations as well, with approximately 26% of all active grants in 2013-2015 lasting 2 years or less; 37% lasting 3-4 years; and 36% lasting 5 or more years.

Whenever start and end dates of grants occur within 2013-2015, they potentially cause the activation or dissolution of grant collaborations during the 3-year period analyzed in the paper. If, for example, a grant involving *i* and *j* starts in 2014, that adds 1 unit in 2014 to the grant collaboration weight between *i* and *j* ($g_{{i,j}_{g,2014}}$). If a grant involving *i* and *j* ends in 2014, that subtracts 1 unit in 2014 to the same grant collaboration weight. In the absence of other grants between *i* and *j*, this causes a grant collaboration between *i* and *j* to be activated or terminated. Similarly, if a peer-reviewed article involving *i* and *j* is published in 2014, that adds 1 unit to the publication collaboration weight between *i* and *j* ($g_{{i,j}_{p,2014}}$), potentially activating a publication collaboration between the two investigators that did not exist before.

When grants and publications are collapsed into union collaborations, the resulting network ties have varied start dates, end dates and durations. This time variation of union collaborations is demonstrated by Fig 5 and Table 8. Only a small proportion of collaborations (2,489 out of 22,171, i.e. approximately 11%, labeled “4” in Fig 5) remain in place throughout the 3-year period analyzed in the paper. Collaborations labeled “1”, “3”, and “6” in Fig 5, which amount to 75% of all collaborations in 2013-2015, are active in one year only (2013, 2014, and 2015, respectively); collaborations labeled “2” and “5” (13% of the total) are active for two consecutive years in the time window (2013-2014 and 2014-2015 respectively). In addition, Table 8 shows that, each year, most active union collaborations either start or end that same year. In 2014, for example, 56% of all active collaborations have started that same year, and 62% end that same year (the two sets overlap, with the intersection consisting of one-year union collaborations that are active in 2014). In each year, only a minority of collaborations remain from previous years or persist in subsequent years. In particular, only 21% of collaborations that exist in 2013 (2,489 out of 11,950) remain active through 2015; and only 30% of all collaborations that exist in 2015 (2,489 out of 8,363) have started in 2013. This indicates that most collaborations in the data appear and dissolve during the selected time window.

Fig 5**. :** Variation of union collaborations

(1) Number of union collaborations that are active in 2013 and end in the same year. (2) Number of union collaborations that are active in 2013 and end in 2014. (3) Number of union collaborations that start in 2014 and end the same year. (4) Number of union collaborations that are active in 2013 and end in 2015. (5) Number of union collaborations that start in 2014 and end in 2015. (6) Number of union collaborations that start in 2015.


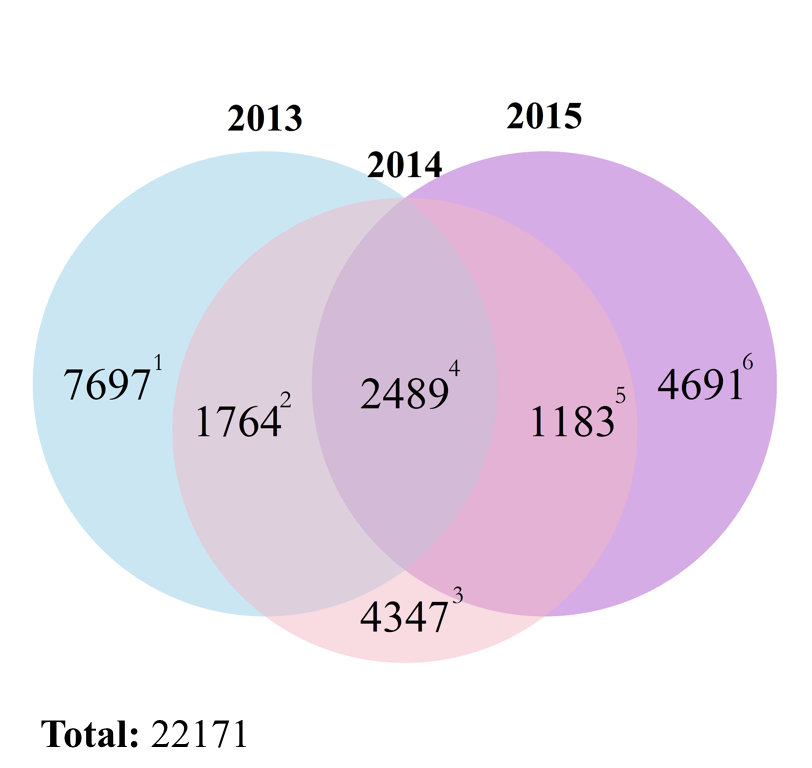


**Table A.** Union collaborations starting and ending each year in cross-sectional union networks.

|  | **Number of union collaborations (% of year’s Total active)** | |
| --- | --- | --- |
| **Cross-sectional network 2013** | Total active | 11,950 |
|  | Ended in 2013 | 7,697 (64%) |
|  | Ended in 2014 | 1,764 (15%) |
|  | Ended in 2015 | 2,489 (21%) |
| **Cross-sectional network 2014** | Total active | 9,783 |
|  | Started in 2013 | 4,253 (44%) |
|  | Started in 2014 | 5,530 (56%) |
|  | Ended in 2014 | 6,111 (62%) |
|  | Ended in 2015 | 1,183 (12%) |
| **Cross-sectional network 2015** | Total active | 8,363 |
|  | Started in 2013 | 2,489 (30%) |
|  | Started in 2014 | 1,183 (14%) |
|  | Started in 2015 | 4,691 (56%) |

## Appendix 2. Structural characteristics of collaborative subgroups and research communities.

This Appendix shows the full cumulative distribution functions of structural characteristics measured on the collaborative subgroups detected in the cross-sectional and overall union networks (see Table 3 for corresponding descriptive statistics), and on the final research communities extracted from the four co-membership networks (see Table 5 for corresponding descriptive statistics).

Fig B**.** Cumulative distribution of main structural characteristics of collaborative subgroups in cross-sectional and overall union networks.


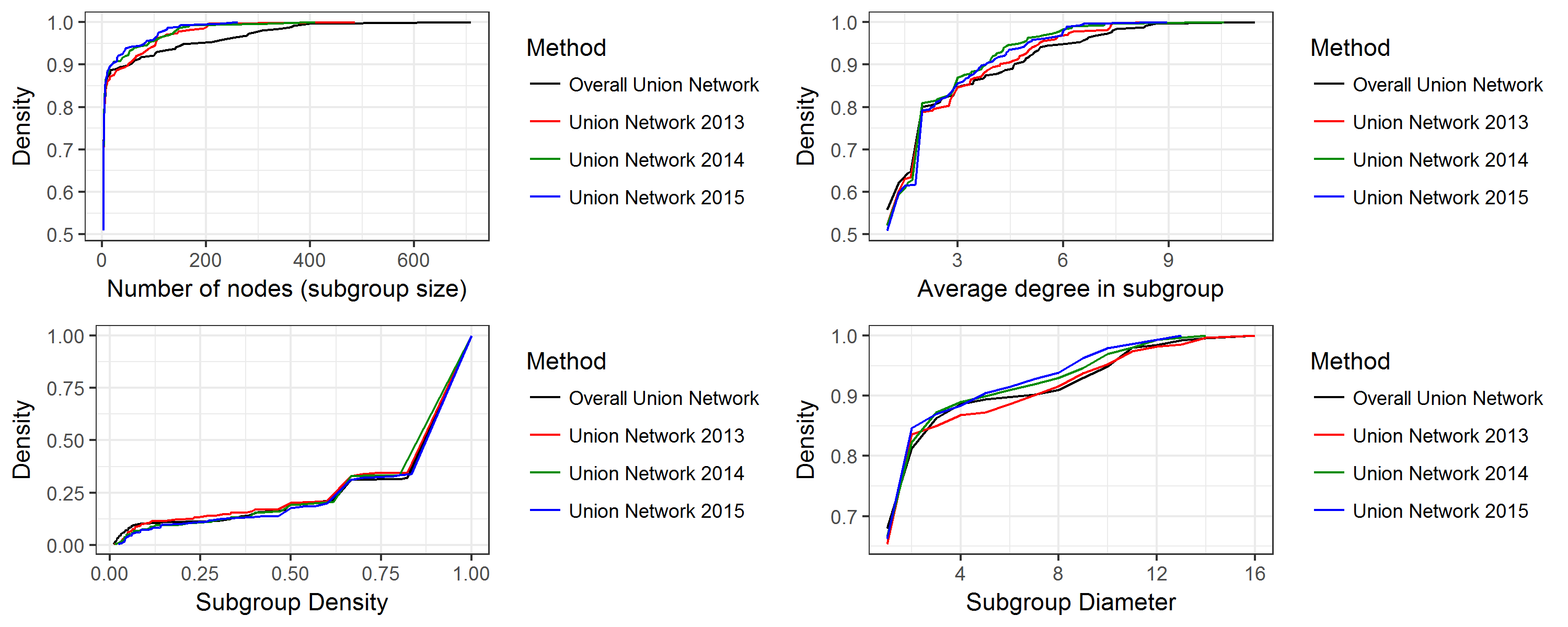


Fig C: Cumulative distribution of main structural characteristics of cross-sectional and intertemporal research communities.


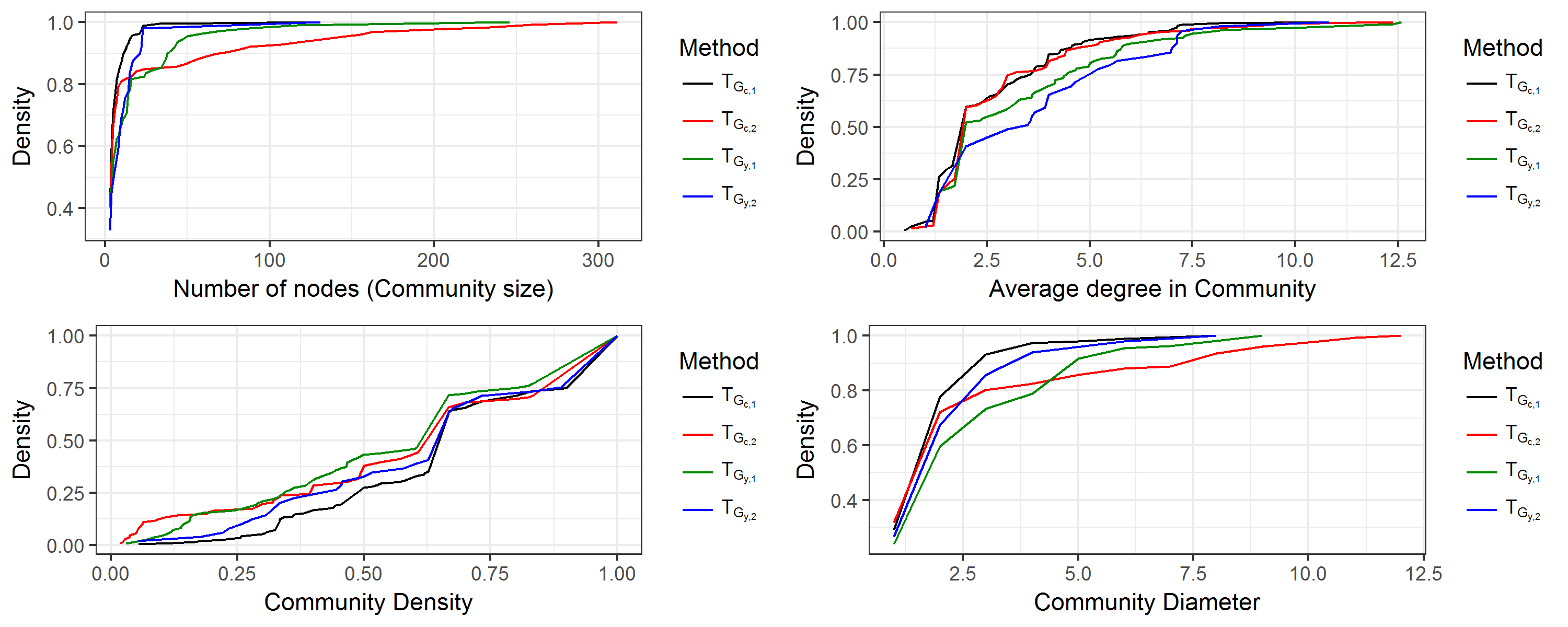

Supplement: S1 File — (DOCX) [file pone.0182516.s001.docx]
